# Unilateral Breast Skin Changes in Patient With Hidradenitis Suppurativa: Malignancy Versus Lymphedema

**DOI:** 10.7759/cureus.16232

**Published:** 2021-07-07

**Authors:** Amanda Ederle, Katie Dreher, Rodrigo Valdes-Rodriguez

**Affiliations:** 1 Internal Medicine, Baptist Health Medical Center-Little Rock, Little Rock, USA; 2 Dermatology, University of Arkansas for Medical Sciences, Little Rock, USA

**Keywords:** hidradenitis suppurativa, lymphedema, inflammatory skin disease, acne inversa, breast surgery, hidradenitis suppurativa complication

## Abstract

Hidradenitis suppurativa (HS) is an inflammatory skin disorder typically affecting the groin, inframammary folds, and axillae. HS is characterized by the development of boils, abscesses, fistulas, and sinus tracts. Due to the inflammatory destruction of lymph vessels, patients with long-standing HS may develop lymphedema. Most commonly reported in the literature is lymphedema involvement of the genital and anal regions. In this case report, we describe unilateral breast skin changes in a patient with HS. The patient was extensively worked up for inflammatory breast cancer, and eventually underwent stereotactic biopsies. Subsequently, these biopsies were consistent with lymphedema due to her chronic HS. Although rare, there is a paucity of literature describing breast lymphedema associated with HS. As breast lymphedema due to HS may mimic inflammatory breast cancer, it is important for providers to firstly rule out malignancy and place lymphedema high on the differential when examining and treating these patients.

## Introduction

Hidradenitis suppurativa (HS) is a chronic, inflammatory skin disorder characterized by painful nodules, abscesses, sinus tracts, and fistulas that typically occur in the groin, inframammary folds, and axillae. Due to the inflammation and scarring, surrounding lymph channels may become blocked, leading to an accumulation of lymphatic fluid in the tissues [1]. Lymphedema is a late, relatively rare complication of HS that classically affects the anogenital region [1-2].

We present a patient with Hurley III HS with a peau d'orange appearance of her breast, raising concern for inflammatory breast cancer. Subsequent biopsies were consistent with lymphedema. Although anogenital lymphedema is a well-documented complication of HS, there appears to be a paucity of literature regarding its occurrence in the breast. However, it is important to recognize it in order to prevent delayed and misdiagnosis or excessive diagnostic testing.

## Case presentation

**Figure 1:**
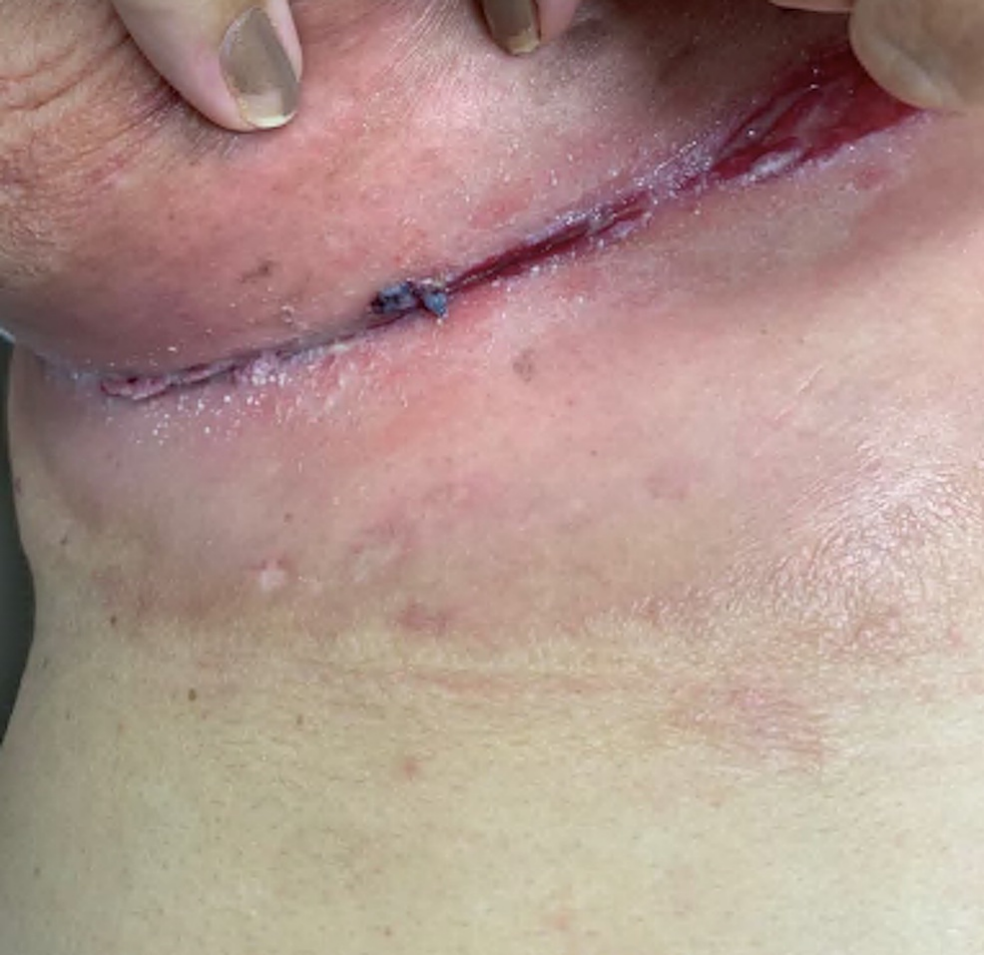
Clinical Image Lymphedema associated with HS. Diffuse erythema of the inferior breast with skin thickening. There are also linear knife-like fissures in the inframammary fold.

A 63-year-old female with longstanding Hurley III HS affecting her bilateral axillae, inframammary region, and groin presented to her primary care physician with a three-month history of unilateral breast redness and heaviness. She denied the presence of any discrete masses or nipple discharge. On physical examination, her breast had ill-defined erythematous and edematous changes with prominent maceration of the lower breast and inframammary fold (Figure 1).

Although she had bilateral HS, her contralateral breast did not exhibit these skin changes. Bilateral axillary scarring was also noted with no actively draining sinuses, focal swellings, or tenderness. Initially, the patient was treated with amoxicillin-clavulanate potassium with suspicion for an infectious etiology. However, there was no improvement of her unilateral breast, making an infectious etiology seem unlikely.

**Figure 2:**
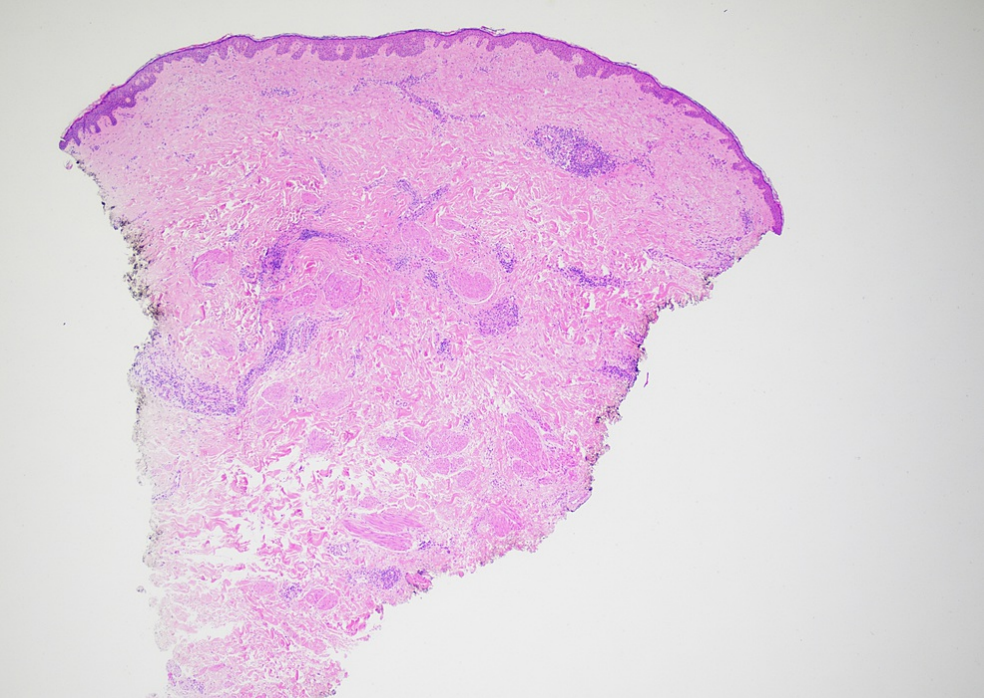
Hematoxylin and eosin stain (20X) Hematoxylin and eosin stain (20X) demonstrating skin with unremarkable epidermis. In the dermis, there is some dermal edema along with some fibrosis and a perivascular inflammatory infiltrate. No malignancy is identified.

Subsequently, the patient underwent a diagnostic mammogram due to concerns for inflammatory breast cancer, which demonstrated skin thickening on the affected breast with suspicious calcifications in both breasts. Bilateral stereotactic biopsies were negative for malignancy or atypia. However, with continued concern for malignancy and worsening of the rash, the patient was sent to a breast surgical oncologist to obtain a punch biopsy of her affected breast. Biopsy specimen at lower magnification showed an uneventful epidermis and perivascular lymphocytic inflammatory infiltrate with mild to moderate dermal edema (Figure 2). 

**Figure 3:**
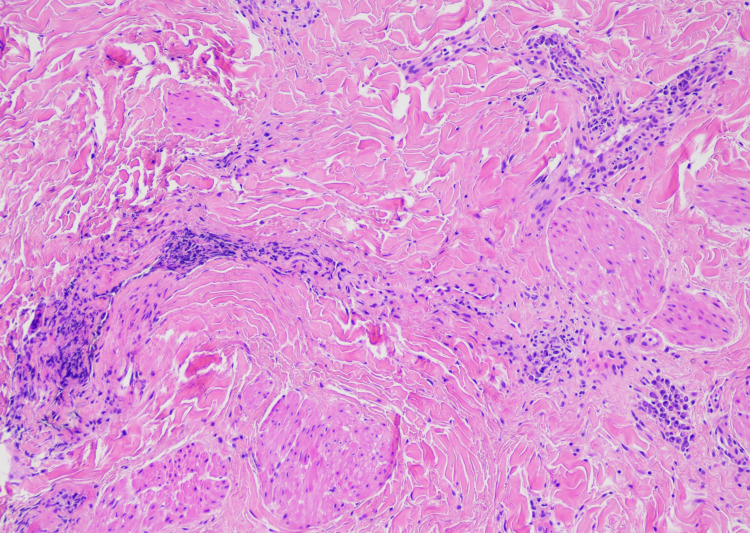
Hematoxylin and eosin stain (100X) Hematoxylin and eosin stain (100X) showing lymphatic vascular ectasia and a perivascular infiltrate composed of lymphocytes, plasma cells. No malignancy is identified.

On high power, there were crack-like vascular spaces surrounded by inflammatory infiltrate composed of lymphocytes and plasma cells (Figure 3). 

Based on the histopathology, these findings were consistent with lymphedema, most likely as a complication of her longstanding HS. The patient was referred back to dermatology for further management of her HS. She was continued on her regimen of adalimumab 40mg weekly and dapsone 25mg daily. She was also advised to wear clothing to support her breasts. A follow-up mammogram was scheduled for four months later which was negative for malignancy in either breast. 

## Discussion

Lymphedema is a rare, yet underdiagnosed complication of longstanding HS [1-3]. The anogenital region is the most common location for lymphedema associated with HS [1]. In a systematic review of lymphedema in patients with HS, 26 out of the 27 patients had involvement of the genitourinary or anogenital region [1]. There has also been one reported case of abdominal lymphedema associated with chronic HS [4]. However, reports of breast lymphedema secondary to HS appear to be lacking in the literature.

As HS frequently involves the inframammary folds and axilla, breast lymphedema associated with HS may be underdiagnosed, causing patients to experience delays in treatment or misdiagnosis. Disruption to the lymphatic channels in this area may cause a build-up of lymph fluid, resulting in clinical skin changes including thickening and pitting of the overlying skin. Since these changes overlap with those of inflammatory breast cancer, it is important to consider this diagnosis and possibly perform further diagnostic studies. Although this may delay the diagnosis of lymphedema associated with HS, ruling out breast cancer should take precedence if a provider is suspicious of cancer. In a patient with long-standing HS, it is imperative to place lymphedema high on the differential diagnosis for a patient presenting with unilateral breast skin changes.

The management of lymphedema associated with HS is, unfortunately, often ineffective. Compression therapy is commonly used as the first line treatment for lymphedema [1,5]. Medical management using adalimumab, infliximab, minocycline, and isotretinoin have also been reported with varying results [1,5,6]. Many providers also treat HS lymphedema with surgical management, which has been effective both functionally and aesthetically [1,7,8].

## Conclusions

This case reports a unique occurrence of unilateral breast lymphedema in the setting of chronic HS, which demonstrates that this HS side effect may be present more broadly than in the anogenital region in which it is classically reported. It is important to distinguish the clinically similar and potentially deadly diagnosis of inflammatory breast cancer from this condition. Once a diagnosis of lymphedema is confirmed, providers can begin to manage these patients more effectively to prevent further progression and morbidity. 
